# Active PLA Packaging Films: Effect of Processing and the Addition of Natural Antimicrobials and Antioxidants on Physical Properties, Release Kinetics, and Compostability

**DOI:** 10.3390/antiox10121976

**Published:** 2021-12-11

**Authors:** Adrián Rojas, Eliezer Velásquez, Cristian Patiño Vidal, Abel Guarda, María José Galotto, Carol López de Dicastillo

**Affiliations:** 1 Packaging Innovation Center (LABEN), Department of Science and Food Technology, Faculty of Technology, University of Santiago of Chile (USACH), Obispo Umaña 050, Santiago 9170201, Chile; adrian.rojass@usach.cl (A.R.); eliezer.velasquez@usach.cl (E.V.); cristian.patino@usach.cl (C.P.V.); abel.guarda@usach.cl (A.G.); maria.galotto@usach.cl (M.J.G.); 2 Center for the Development of Nanoscience and Nanotechnology (CEDENNA), Santiago 9170124, Chile; 3 Department of Science and Food Technology, Faculty of Technology, University of Santiago of Chile (USACH), Obispo Umaña 050, Santiago 9170201, Chile

**Keywords:** PLA, active food packaging, antimicrobial, antioxidant, physical properties, release kinetics, compostability

## Abstract

The performance characteristics of polylactic acid (PLA) as an active food packaging film can be highly influenced by the incorporation of active agents (AAs) into PLA, and the type of processing technique. In this review, the effect of processing techniques and the addition of natural AAs on the properties related to PLA performance as a packaging material are summarized and described through a systematic analysis, giving new insights about the relation between processing techniques, types of AA, physical–mechanical properties, barriers, optical properties, compostability, controlled release, and functionalities in order to contribute to the progress made in designing antioxidant and antimicrobial PLA packaging films. The addition of AAs into PLA films affected their optical properties and influenced polymer chain reordering, modifying their thermal properties, functionality, and compostability in terms of the chemical nature of AAs. The mechanical and barrier performance of PLA was affected by the AA’s dispersion degree and crystallinity changes resulting from specific processing techniques. In addition, hydrophobicity and AA concentration also modified the barrier properties of PLA. The release kinetics of AAs from PLA were tuned, modifying diffusion coefficient of the AAs in terms of the different physical properties of the films that resulted from specific processing techniques. Several developments based on the incorporation of antimicrobial and antioxidant substances into PLA have displayed outstanding activities for food protection against microbial growth and oxidation.

## 1. Introduction

Environmental pollution generated from the accumulation of plastic waste is undoubtedly one of the most urgent environmental problems that needs to be addressed. This fact originates mainly due to the increase in disposable plastic products principally used in the food packaging sector, whose degradation time can extend several hundred years. An alternative to these long-lasting synthetic plastics is biodegradable materials, where polylactic acid (PLA) has become the most commonly used environmentally friendly bioplastic because it is commercially available, compostable, and can be processed by conventional melting extrusion [1,2]. PLA is a biopolymer that has received increasing attention in the food packaging industry in recent decades due to its sustainability, biocompatibility, biodegradability, and safety [3,4]. PLA is a linear aliphatic thermoplastic polymer produced by the polymerization of L- and D- lactic acid isomers, which are obtained through the fermentation of starch from renewable sources such as potatoes, sugarcane, and corn [5]. Meanwhile, at an industrial level, PLA is primarily produced through the polycondensation of lactic acid and/or the ring-opening polymerization of lactide [6]. Due to its high transparency, easy processability, resistance to chemicals, and good and smooth appearance, PLA has been processed for industrial film production, mainly by extrusion, casting, and blow-molding. Nevertheless, PLA packaging films have also been obtained through other techniques such as compression molding, solvent casting, and electrospinning, among others [7].

An interesting research area in PLA food packaging is the development of active release systems [8]. Active release packaging is an innovative concept, which involves the interaction between package and food through the release of active substances from the polymer matrix to the food surface in order to prolong shelf life or enhance its safety and/or sensory properties, while simultaneously preserving the quality. One of the biggest challenges of active food packaging is providing superior protection against microbiological deterioration and oxidative reactions compared to conventional food packaging. Incorporating AAs inside the polymer matrix films allows for their functionalization. In this context, the primary deterioration process of the packaged food product, such as the oxidation process, or bacterial and fungal incidence, determines the selection of the active agent. Natural extracts and essential oils (EOs) from plants or their derivatives have been used mainly to develop antimicrobial and antioxidant activities due to their recognition as safe [9]. The characteristics of EOs as food ingredients or as part of the packaging have recently been reviewed [10,11]. AAs have been incorporated into the polymer structure during the film formation through melt-blending processes, such as extrusion and compression-molding processes [12], or through other techniques, such as solvent-casting [13] and electrospinning processes [14], where PLA was previously dissolved, and lower temperatures were needed. An alternative strategy to develop active PLA packaging films has also been carried out by incorporating thermosensitive compounds into the PLA matrix by a supercritical carbon dioxide (scCO_2_) impregnation process [15]. The feasibility, advantages, and limitations of the current processing techniques used to develop active PLA packaging films have recently been reviewed [16].

The addition of an AA to PLA changed its physical properties, i.e., its barrier, mechanical, thermal, and optical properties. Several works have reported that AAs that exhibited nucleating and/or plasticizing properties sometimes modify the crystallinity degree of PLA and its physical–mechanical characteristics, compostability, and mass transfer properties. In this context, several studies have been carried out to evaluate the effect of AAs, such as thymol, eugenol, and some natural extracts, on the thermal, optical, barrier, mechanical, and biodegradability properties of PLA [17,18,19,20].

Furthermore, the functionality analysis of these active PLA packaging films has essentially been determined through in vitro assays following standard procedures and methods [21,22]. The effectiveness of AA-modified PLA films depends on the release kinetics of the AA from the polymer to the food, which results from the mass transfer phenomena that involve the equilibrium and kinetic processes in both food and packaging material. Although few studies have dealt with the assessment of the release kinetics of AAs from PLA films, results have shown that the release process was dependent on the processing technique and the AA’s chemical nature [12,15].

Previous works have already compiled the description of antioxidant and antimicrobial PLA film developments due to the relevance of PLA in the design of bioderived active packaging [23,24,25]. In this framework, this review was focused on summarizing the effect of processing techniques and the addition of AAs on the main physical and compostability properties of PLA in a critical and comprehensive manner. Additionally, their functionalities for food packaging applications and their mass-transfer properties for AA release were analyzed. From the described features, this review gives new insights on the relation between processing techniques and the type of AA, physical–mechanical properties, compostability, and controlled release from active PLA packaging films in order to contribute to the progress of designing antimicrobial and antioxidant PLA packaging films with desired characteristics for specific food packaging applications.

## 2. Effect of Processing and Incorporation of Active Agents in Physical–Mechanical Properties

### 2.1. Optical Properties

Color and opacity are two of the main optical properties to be evaluated during the development of active films, since noticeable changes in these parameters could negatively affect the consumer’s acceptability towards their application as food package materials [26]. PLA can be produced with high transparency, and mono- or several multilayer PLA films maintained this character regardless of the processing technique [7,27,28].

Regarding color changes on PLA-based films, the intrinsic color of an AA and/or the polymer were the main factors that produced such variation, and it was generally expressed as ΔE. A slight change in ΔE value of ±0.5 can be recognized by humans as a different color, therefore producing a rejection of the food which was packaged. Furthermore, this effect can be intensified if ΔE is higher than a value of 2.0 [29]. On the other hand, the transparency of films has been related to the capability of light transmission through the material, which is typically measured by spectrophotometric methods at visible wavelength values between 500 and 600 nm. The opacity and transparency are inversely proportional parameters and depend on the film’s thickness [13,30]. Maintaining the film’s transparency after incorporating an AA is highly preferred in active packaging design when being able to observe the food is desirable. On the other hand, the increase in the opacity of PLA packaging films can be significant for avoiding light-induced degradation processes, such as fat oxidation and pigment degradation. Opacity has been attributed to the differences in the refractive indices of the AA and PLA matrix, which modify the light scattering and absorption through the film and, therefore, the light transmittance [30].

Some works have shown a color change in PLA films when the AA has been directly added (non-encapsulated AA). For example, the incorporation of *Allium ursinum* extract (AU) (wild garlic) into solvent-cast PLA films displayed ΔE values of 4.4 and 41.4 when AU was added at 0.5 and 5 wt% in PLA, respectively, resulting in transparent and yellowish films [31]. Likewise, the intrinsic color of freeze-dried orange peel extract directly added to PLA, or previously encapsulated with either pectin or β-cyclodextrin (CD) through spray drying, and subsequently incorporated in the polymeric matrix, modified the color of PLA films obtained by hot pressing [32]. Freeze-dried extract and spray-dried powder at concentration values between 0.25 and 2 wt% were loaded into PLA films. Researchers reported a more intense orange coloration of PLA as the concentration of the extracts were increased.

Similarly, certain polymers used in PLA-based bilayer films or in AAs incorporated in some of the layers have also produced a color variation of the films. The application of a zein coating loaded with quercetin at 5 wt% onto an extruded PLA film changed its color, but maintained its transparency [33]. The resulted bilayer films reflected the characteristic yellowish tone from both zein and quercetin incorporation. The PLA/zein bilayer displayed ΔE values of 1.95, which was increased to 5.36 when quercetin at 5.0 wt% was incorporated into the zein layer. Likewise, a recent study about PLA-based bilayers evidenced an increase in ΔE value toward yellowness when a gelatin layer loaded with different concentrations of epigallocatechin gallate (EGCG) (0–12 wt%) was incorporated into PLA through compression molding [34]. Furthermore, the films showed a good transparency. The incorporation of umbelliferone (UMB) also produced a red-yellowish coloration in mono- (PLA) and bilayer (PLA/PLA) films obtained through extrusion and compression molding techniques [35]. The active PLA films showed high ΔE values with respect to their corresponding monolayer (ΔE = 23.9) or bilayer (ΔE = 58.4) controls. On the other hand, the low miscibility between AAs and the polymeric matrix due to their different chemical characteristics can intensify the opacity of the films [36,37]. This effect can be intensified at high AA loads since a good dispersion of AA into PLA films is hindered. This fact results in the formation of particles larger than the visible wavelength, blocking the light path and increasing the opacity of films. A study about an active PLA film loaded with clove essential oil at 15 and 30 wt%, and graphene oxide nanosheets at 1 wt%, clearly evidenced this effect [13]. The increase in the loading of EO reduced the light transmittance, and this reduction was intensified by the presence of graphene oxide nanosheets. Researchers attributed this drop to the interruption of light passage by the nanosheets and to a bad dispersion of oil droplets in the polymer matrix. Likewise, a work based on PLA active films loaded with β-cyclodextrin/2-nonanone inclusion complexes at 5 and 10 wt% also showed a similar behavior. The incorporation of inclusion complexes increased the opacity of PLA active films up to 3-fold due to the presence of agglomerates which scattered light and increased opacity [37]. The immiscibility between the inclusion complexes and PLA increased the film discontinuities and films’ opacity, and this effect increased as the concentration of the inclusion complex containing AA increased.

### 2.2. Thermal Properties

Semi-crystalline polymers, such as PLA, show several thermal transitions when heated as endothermic processes, such as glass and melting transitions, and exothermic processes, such as thermo-oxidative degradation, crosslinking reactions, and crystallization. Furthermore, heating also induced the cold crystallization of PLA, which is associated with the transition from an amorphous to a crystalline phase, including the rearrangement of imperfect crystals. Heating also induced polymer changes from a glassy solid state to a viscoelastic rubbery state, with greater mobility above the glass transition temperature (T_g_) associated with the amorphous zones. Subsequently, the crystals collapse at the melting temperature (T_m_). The values of these temperatures enable the evaluation of the conditions of processability of PLA films for different applications.

Thermal parameters of PLA and active PLA films reported by several works are summarized in Table 1. PLA presented the glass transition at around 60 °C and the melting transition at between 145 and 170 °C. Lower T_g_ values were reported for PLA obtained by electrospinning or solvent casting, possibly due to the presence of residual solvent that acts as a plasticizer. These thermal parameters depended on several factors associated with the grade of PLA (L- and D-lactide content, melt flow index, molar mass, and dispersity index) and the processing conditions [31,38,39]. The thermal transitions of PLA, manifested as heat flow changes with the temperature variation, were detected through differential scanning calorimetry (DSC) analysis. Moreover, the crystallinity has been calculated by the ratio between the melting heat of the PLA sample and the melting heat of 100% crystalline PLA [40,41]. The effect of the processing technique on the PLA’s thermal properties has been evaluated by the first DSC heating process. In this context, significant differences have mainly been found between extruded and electrospun PLA films obtained using virgin PLA of the same grade. The T_g_ of PLA films obtained by electrospinning tended to be at lower values (40 °C) [14] than the T_g_ of PLA films obtained through extrusion (61 °C) which can be associated with the residual solvent in the PLA [37]. Meanwhile, lower crystallinity has been reported for electrospun PLA films than extruded PLA films, as a result of the inhibition of the polymer chain order due to fast cooling produced by solvent evaporation and the large surface area of the nanofibers [42].

PLA’s thermal parameters also depended on the processing conditions and/or measurement parameters of the DSC analysis, which could be responsible for the differences found for the T_m_ and X_c_ of extruded PLA [37,42].

Regarding PLA films obtained by electrospinning, lower values of T_g_ and T_m_ (40 °C and 137 °C) were reported for PLA films obtained using CHCl_3_:DMF at 7:3 volume ratio [14] instead of CHCl_3_:DMF at 1:1 solution (53 °C and 153 °C), using PLA with the same grade [42]. These results suggest a higher volatility of chloroform-inhibited chain ordering during fiber formation when CHCl_3_:DMF ratio 7:3 was used, so the polymeric film was more amorphous, and therefore flexible, with a lower melting temperature. Furthermore, it is important to highlight that different amounts of residual solvent in the fibers could cause different plasticization effects and T_g_ values. Similarly, solvent volatility and casting conditions strongly influenced the thermal parameters of cast PLA films [44,45].

Thermal parameters of active PLA films reported by several works are summarized in Table 2. The incorporation of AAs has modified the thermal properties and crystallinity of PLA in terms of the AA chemical structure. The PLA–AA interactions increased T_g,_ and caused a reduction of the crystallinity due to the changes in the mobility of polymeric chains during the processing of PLA-curcumin by electrospinning [14]. This effect was attributed to hydrogen-bond interactions between PLA and the curcumin. Furthermore, the curcumin caused PLA to cold crystallize during first heating process, promoting a reordered structure with a unique melting transition [14]. Conversely, some AAs exerted a plasticizing effect on the PLA chains by lowering their glass transition and melting temperatures, as observed by incorporating ungeremine into PLA by electrospinning [46,47].

Nonetheless, the changes in PLA crystallinity depend on the AA concentration. Radusin et al. (2019) [31] reported that wild garlic at low concentration exerted a nucleating effect on cast PLA films due to the good dispersion of this antimicrobial compound. Conversely, the formation of amorphous zones was favored at higher AA concentrations due to polymer-chain-ordering disruption through steric hindering [31].

Furthermore, it is highlighted that the molecular structure of the AA plays a fundamental role in the formation of crystals by processing techniques involving stretching. For example, a 14% reduction in PLA crystallinity was reported, with the addition of 20 wt% thymol, in PLA films obtained by extrusion and biaxial stretching. In contrast, the degree of crystallinity increased by 7.3% when R-(−)-carvone was added using the same technique at the same nominal concentration, considering that final concentration in the films was reduced to lower than 50 wt% of the initial concentration loaded. These results suggested that orientation- and strain-induced crystallization in films containing R-(−)carvone could be reached [48].

On the other hand, some works have evaluated the thermal properties of active PLA films after erasing the thermal history of the polymer through a second heating process to study the effect of the concentration and chemical structure of the AA, which modifies the PLA chain ordering. A drop of 3 °C in the T_g_ of cast PLA films was reported, due to the incorporation of bergamot essential oil at 9 wt%, and a 6 °C reduction due to clove oil added at the same concentration [49]. Meanwhile, a more significant temperature drop in the T_g_ of extruded PLA films was reported when the concentration of thymol in the PLA films was increased from 5 wt% (5 °C) to 20 wt% (26 °C) [38].

The thermal stability of PLA was modified by the incorporation of AAs in terms of their molecular structure. Gavril et al. (2019) [50] reported a drop of 10 °C and 13 °C in temperature at the maximum degradation rate of PLA films (T_d_ = 345 °C) obtained by melt mixing with lemon balm leaves and sage at 3 wt%, respectively. Some active PLA films obtained by different types of processing have evidenced two degradation processes: (i) the first stage, corresponding to the decomposition of the AA; and (ii) the second stage, associated with PLA degradation at higher temperatures [15,49,51]. Nonetheless, antioxidant compounds such as cinnamaldehyde (CIN) and curcumin have improved the thermal stability of PLA [14,42]. Moreover, the encapsulation of AAs such as carvacrol, thymol, and 2-nonanone in β-cyclodextrin and pectin, has effectively inhibited the degradation of the EO contained in PLA matrices [37,52].

### 2.3. Mechanical Properties

PLA is a rigid low-stretch polymer with mechanical properties comparable to those of poly(ethylene terephthalate) (PET) and polystyrene (PS), although PLA is more brittle and less impact-resistant [53]. Mechanical properties depend on PLA architecture based on L, D, and meso-lactide proportions, molar mass, and processing conditions [54]. The ratio of lactide isomers influenced the thermal properties and the crystallinity, and therefore, the flexibility of the PLA film.

The mechanical performance of PLA is commonly evaluated by tensile testing that reveals its main mechanical parameters: (i) Young’s modulus (YM): the slope of the elastic-linear zone in the stress vs. strain curve and the measuring of the stiffness; (ii) tensile strength (TS): the maximum force the material can withstand; and (iii) elongation at break (EB): material extensibility.

Table 3 shows the tensile parameters of active PLA films obtained through different processing techniques. In general, the YM, TS, and EB values of PLA films were reported as between 2800–3400 MPa, 40–65 Mpa, and 1–6%, respectively. Exceptionally, the YM of solvent-cast PLA films (approx. 1700 MPa) has shown a value approximately 50% lower than the YM values of PLA films obtained through melting processes (Table 3). Similarly, electrospun PLA films have exhibited lower stiffness and tensile strength depending on the fiber diameter, and high levels of deformation at break (approx. 60%) (Table 3). This fact may be associated with the lower crystallinity degree of electrospun PLA films compared with extruded PLA films [14,37,42].

The effect of the addition of AAs on the mechanical properties of PLA films depended on the molecular structure of the AA, the processing technique, and the conditions. Table 3 shows the mechanical properties of recent active PLA developments. The changes in the mechanical properties of PLA films were greater as AA concentration increased. In general, YM values of PLA films decreased with AA incorporation. This fact is mainly attributed to a plasticizer effect of the AA, which was located between PLA chains. This increased the free volume, reduced chain entanglements, and caused greater chain mobility and flexibility. On the contrary, when chitosan was added to PLA, this AA acted as a reinforcing filler and restricted the segmental chain movement of PLA. The stiffness of the film increased due to the rigidity of these polysaccharide chains and the generated interactions between PLA and chitosan [55]. These interactions could be hydrogen bonding between PLA (carbonyl or end hydroxyl groups) and hydroxyl, and amine/acetamide groups of chitosan [56]. Nonetheless, the Young modulus of PLA films with chitosan at concentrations above 10 wt% decreased due to the formation of chitosan agglomerates [55]. Moreover, TS variation was attributed to the concentration and dispersion of the AA in the PLA matrix. TS of the films increased with chitosan loaded at 5 wt%, but decreased gradually with up to 15 wt% of chitosan. This gradual reduction was associated with the formation of chitosan aggregates surrounded by a weak interface that acted as stress concentrator lowering tensile strength [55].

Other works have reported that the AA addition has led to a reduction in TS values, possibly due to lower PLA–PLA intermolecular forces generated by the intercalation of AA molecules between the polymer chains (Table 3) [13]. Meanwhile, some studies have reported increases in TS values for active PLA films. For example, increases of 95% and 48% were reported when AU extract (wild garlic) and propyl gallate were incorporated in cast PLA films and in the core of a coaxial PLA/PLA fiber, compared to the uniaxial PLA fiber, respectively [31,57]. Furthermore, in the case of PLA fibers, TS depended on the fiber diameter which varied with the PLA solution’s viscosity, and therefore with the AA concentration. In this context, Liu et al. (2018) [58] reported a decrease in the TS values of PLA films due to the addition of tea polyphenols, which decreased PLA solution viscosity and thus reduced the fiber diameter.

Regarding the elongation at break of active PLA films, several works have reported a plasticizing effect of the AA due to its intercalation between the polymer chains that caused higher molecular mobility and film deformation (Table 3). In extrusion-stretching processes, the ductility of PLA films was increased according to the chemical structure and concentration of the AA. Martins et al. (2018) [60] reported the EB’s increase of blown extruded PLA films by 9.6% and 36% due to the addition of green tea (GT) extract at 1.0 and 2.0 wt%, respectively. Similarly, Boonruang et al. (2016) [48] reported that thymol and R-(−)-carvone increased the extensibility of PLA films obtained by extrusion-biaxial stretching (BOPLA). Although both AAs presented a plasticizing effect, the magnitude of the EB variation depended on the changes in crystallinity of the resulted films according to the chemical structure of the AA. Furthermore, thymol exhibited a dramatically more significant plasticizing effect for injection-molded PLA than PLA films obtained by other techniques, such as melt blending, hot pressing, extrusion, and biaxial stretching. Celebi and Gunes (2018) [38] reported that the EB of injection-molded PLA containing 20 wt% of thymol was 144 times higher than that of the neat PLA, while Boonruang et al. (2016) [48] reported a 3.6-fold increase at the same thymol nominal concentration compared to neat PLA films processed through extrusion stretching. This comparison is made based on the initial AA load added to PLA and does not consider the loss of AA associated with each process, which is unknown for the injection-molded PLA.

Nonetheless, some studies have reported that EB values decrease for active PLA films due to different factors. Vasile et al. (2019) [55] pointed out that the addition of chitosan at 6 wt% reduced the ductility of PLA films melt-blended with polyethylene glycol (PEG) as a plasticizer. However, the simultaneous addition of chitosan and rosemary ethanolic extract drastically increased the extensibility of PLA by up to 52% due to a synergistic effect and interactions between both components. On the other hand, it was observed that incorporating *Allium* extract at 2 wt% reduced the EB value of extruded PLA films by approximately 16% due to the addition of these AA produced films with lower thickness and homogeneity [51]. Similarly, the incorporation of merkén, a Chilean spice containing antioxidant activity, decreased EB values due to discontinuities in the extruded PLA films [43].

### 2.4. Moisture and Gas Barrier Properties

**Figure 1:**
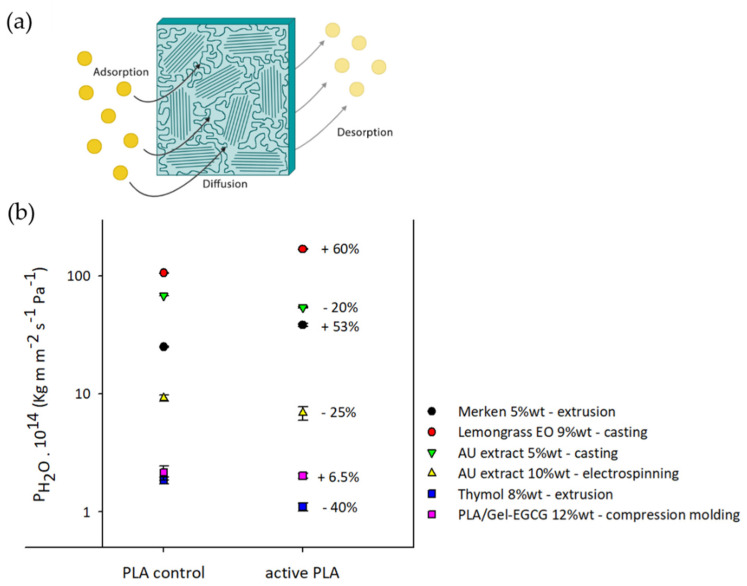
(**a**) Diagram of the mechanism of gas or water vapor permeation through a plastic film; (**b**) Increase and/or decrease of water vapor permeabilities, expressed as %, due to AA incorporation found in different works: Merkén [43]; Lemongrass essential oil [49]; *Allium ursinum* L. extract [31]; *Allium ursinum* L. extract [65]; Thymol [66]; and bilayer structure PLA/Gelatin containing epigallocatechin gallate [34].

Barrier properties, including permeability to gases—such as O_2_, CO_2_, and aroma compounds—and water vapor transmission are crucial parameters in the development of food packaging films. The permeability of polymers results from the sorption process of permeant molecules followed by their diffusion through the polymeric matrix [63]. Specifically, because of the semi-crystalline nature of PLA, their barrier properties are closely affected by the film crystallinity, since the diffusion mechanisms of these molecules occur through the amorphous regions [64], as shown in Figure 1a.

The barrier properties of PLA films were extensively studied, and in general terms, these values were medium [54]. Specifically, CO_2_ permeability coefficients, P_CO2_, for PLA (approx. 0.7 × 10^−17^–1.1 ×10^−17^ kg m m^−2^ s^−1^ Pa^−1^) were lower than for crystal polystyrene, PS (1.55 × 10^−16^ kg m m^−2^ s^−1^ Pa^−1^), but higher than those for polyethylene terephthalate, PET (1.7 × 10^−18^–3.17 × 10^−18^ kg m m^−2^ s^−1^ Pa^−1^) [54]. The oxygen permeability coefficient, PO_2_, follows the same trend with values around 20 times lower than those published for PS, but higher than those reported for PET. These values depended on temperature and were ranged between 1.2 × 10^−18^–4 × 10^−18^ kg m m^−2^ s^−1^ Pa^−1^ [54]. The effect of the relative humidity (RH) on oxygen barrier properties were also analyzed, and the values followed a similar behavior to the PET polymer. Although oxygen diffusion coefficients increased with water activity, P_O2_ values did not change significantly [67]. The incorporation of AAs and plasticizers affected the barrier properties of PLA since permeability values depended on intrinsic factors, such as PLA architecture (L, D isomeric composition), crystallinity degree, thickness, free volume, and chain stiffness. On the other hand, extrinsic factors included processing technique and conditions, e.g., temperature and relative humidity [68]. It is essential to consider the difficulty in establishing correlations and comparisons between different active PLA films, because many investigations did not include data on properties such as thickness or crystallinity, and because the units of permeability were not standardized. Nonetheless, Table 4 summarizes some works that present the effect of AA addition on the moisture and oxygen permeability (P_H2O_ and P_O2_) of active PLA films obtained through different processing techniques. Several works evidenced that the variation in oxygen permeability was highly dependent on the concentration of AA and the crystallinity degree of active materials. The crystallinity degree decrease due to the addition of AAs was proportional to the increase of gases and moisture permeability values in active PLA films [13,43].

When AAs were added at low concentrations, barrier properties were not generally affected, and in some cases, they were improved. P_O2_ values of solvent-cast PLA films containing allyl isothiocyanate at 5 wt% decreased from 3.77 × 10^−6^ to 2.3 × 10^−6^ m^3^ m^−2^ s^−1^ [44]. On the other hand, extruded PLA films containing thymol and carvone at concentrations between 10 and 20 wt% presented gas permeability behaviors dependent on the concentration and the type of these AAs. As Table 4 shows, the addition of both AAs at 10 wt% improved the oxygen and carbon dioxide barrier properties of the extruded PLA films. Nevertheless, P_O2_ and P_CO2_ values were increased greatly at higher concentrations. Active PLA films with thymol at 20 wt% revealed the highest value of P_O2_, because thymol acted as a plasticizer and rearranged the polymer chains layers, lowering the degree of crystallinity and increasing the free volume in the polymer matrix. On the other hand, PLA film containing 20 wt% of carvone manifested the highest P_CO2_ value due to the higher solubility of CO_2_ in this AA [48]. Generally, incorporating AAs at high concentrations (approx. 10–30 wt%) entailed an increase in oxygen permeability due to the increase of the O_2_ diffusivity in the film. The increase in oxygen diffusion at high AA concentrations could also be a result of the increase in the free volume of the polymer in the interphase between the AA and the PLA matrix, probably due to the formation of discontinuous phases [69].

The same tendency was observed by solvent-cast PLA films containing AU extract at 0.5 and 5 wt%, whose P_O2_ values were influenced by AU concentration and relative humidity (RH). In dry conditions (0% RH), the PLA barrier properties were improved slightly by the addition of AU at both concentrations. Higher crystalline content in active PLA films positively influenced its barrier properties. Nevertheless, in humid conditions (50% RH), both active films presented different behaviors. PLA with a low AU concentration exhibited lower oxygen permeability than that of pure PLA, while P_O2_ significantly increased at a high AU concentration [31]. In this case, it was evidenced that, unlike pure PLA, RH affected the gas barrier properties of active PLA films, principally when the AA displayed moderate hydrophilicity due to the interaction between the AA and water vapor.

The incorporation of plasticizers, such as tributyl citrate (TBC), also affected the barrier properties of PLA films in some developments [44]. The water vapor permeability values of PLA film increased from 2.06 × 10^−6^ to 2.38 × 10^−6^ kg m^−2^ s^−1^ with the incorporation of TBC at 7.5 wt%, due to the free volume generated by the addition of plasticizer [44].

In conclusion, the addition of AAs did not present a unique tendency in their effect on the oxygen barrier performance of active PLA films. The gas permeability would be dependent on the AA concentration and the crystallinity of the resulted active films, but also on the hydrophilic character of the AA, and even the relative humidity.

With regard to water vapor barrier properties, in general, although PLA is a polar material, this polymer presented interesting water vapor permeability coefficients (WVPC) [54]. The effect of temperature and RH in two extruded PLA films was analyzed, and evidenced that WVPC was maintained reasonably stably as RH was increased. Meanwhile, a decrease in WVPC values was observed as the temperature was increased [54]. In this study, the reported PLA WVPC values ranged from 2.2 × 10^−14^ to 1.48 × 10^−14^ kg m m^−2^ s^−1^ Pa^−1^ when temperature increased from 10 to 37.8 °C, while PET and PS films exhibited values of approximately 1.1 × 10^−15^ and 6.7 × 10^−15^ kg m m^−2^ s^−1^ Pa^−1^, respectively (at 25 °C).

Figure 1b summarizes some results presented in various developments of active PLA films obtained through different processing techniques, with the intention of observing the effect of incorporating an AA on WVPC values. Figure 1b also shows the increase or decrease in WVPC results, expressed as % with respect to the neat PLA. In general, no specific trend in WVPC of PLA was found with the incorporation of AAs, nor by using a specific processing technique. In some cases, WVPC values of PLA depended on the AA type and their concentrations. The addition of an AA at low concentrations has resulted in either a WVPC increase or decrease with respect to the neat PLA. As Table 4 shows, incorporating GT at 1 and 2 wt% has improved the water barrier properties of extruded PLA films, decreasing WVPC values from 9.31 × 10^−7^ to 6.06 × 10^−7^ kg m^−2^ s^−1^ [60]. Meanwhile, as Figure 1b shows, an extruded PLA film containing 5 wt% of merkén, a Chilean spice with antioxidant properties, exhibited an increase of WVPC from 2.5 × 10^−15^ to 3.83 × 10^−15^ kg m m^−2^ s^−1^ Pa^−1^ [43]. Although merkén contains hydrophobic antioxidant compounds, such as rutin, myricetin, and quercetin, this spice consists of high levels of organic matter content, since it is made from dry and crushed Chilean peppers. This organic fraction possibly hindered the miscibility of antioxidant compounds with PLA. Merkén components at both 3 and 5 wt% decreased PLA crystallinity (see Table 4) and increased its free volume; therefore, the polymer chain mobility and the molecular diffusivity were increased. Moreover, the formation of agglomerates could also have influenced permeation due to the generation of spaces in the interfacial zones. Thus, the effect of the AA’s addition on PLA crystallinity and its dispersion into the polymer matrix are crucial factors to be considered [43].

When the AA was incorporated at high concentrations, an increase or decrease of permeability with respect to the neat PLA was also displayed. Different Eos, such as bergamot, lemongrass, rosemary, and clove oil were incorporated into PLA at 9 wt% through the solvent-casting process. All samples revealed an increase of WVPC values from 1.06 × 10^−14^ kg m m^−2^ s^−1^ Pa^−1^ to 1.7–1.9 × 10^−14^ kg m m^−2^ s^−1^ Pa^−1^ (Figure 1b) [49]. Conversely, some studies also showed improved water vapor barrier properties of active PLA films (Figure 1b). Solvent-cast active PLA films containing propolis extract and powdered raw propolis at 8.5 and 13 wt% manifested lower WVPC values (approximately 1.36 × 10^−14^ kg m m^−2^ s^−1^ Pa^−1^) than PLA control film (2.39 × 10^−14^ kg m m^−2^ s^−1^ Pa^−1^), and the improvement was proportional to propolis concentration, probably due to the high hydrophobicity of these AAs [61]. Recently, cast PLA films containing thymol, kesum, and curry at 10 wt% evidenced a decrease in WVPC values from 4.35 × 10^−18^ kg m^−1^ s^−1^ Pa^−1^ to 3.97 × 10^−18^, 3.83 × 10^−18^ and 3.85 × 10^−18^ kg m^−1^ s^−1^ Pa^−1^, respectively [72]. Although Mohamad et al. (2020) stated that the increased barrier to the diffusion of water molecules into the polymeric matrix was attributed to the strong interactions between PLA and the AA through hydrogen bonding, the hydrophobicity of these AAs also had a barrier effect against water vapor permeability. This fact was in accordance with works that had also evidenced an increase in the contact angle of PLA by the addition of hydrophobic AAs [73].

Although the analysis of the barrier properties of active electrospun PLA films is not very common, Radusin et al. (2019) demonstrated that incorporating AU extract at 10 wt% through electrospinning decreased the WVPC values by approximately 25% (from 9.2 × 10^−14^ to 6.9 × 10^−14^ kg m m^−2^ s^−1^ Pa^−1^) (see Figure 1b) [65]. In general, electrospun active PLA mats are expected to display lower barrier properties than PLA films obtained through other techniques due to the action of their higher surface area, fibrillary morphology, and higher porosity [74].

The study of the barrier properties of PLA films after processing with high pressure-CO_2_ has also been very rare. The impact of high-pressure CO_2_ on water vapor barrier properties was analyzed and revealed an increase in WVPC more significant than expected, due to scCO_2_ acting as plasticizer and being physically intercalated between polymer chains, increasing the sites for sorption of water molecules [75]. 30 μm-thick PLA films presented an initial WVPC coefficient of 5.1 × 10^−6^ kg m^−1^ s^−1^ Pa^−1^ and, after CO_2_ sorption at 25 bar, WVPC increased to 7.2 × 10^−6^ kg m^−1^ s^−1^ Pa^−1^.

## 3. Release Kinetics of Active Agents and Their Functionalities

The migration process is the unintended release of additives and degradation compounds from polymers into food. The study of the migration process can be done through: (i) food simulants, in accordance with the European Committee for Standardization [76]; (ii) predictive mathematical models [77]; and (iii) molecular dynamics simulation approaches, based on classical or quantum physics theories [78]. Migration is a mass-transfer process characterized by the rate (release kinetics) and the level of migration (thermodynamic equilibrium) that are determined by the properties of the polymer (crystallinity, crosslinking degree, etc.), physical and chemical properties of the migrant and the food, type of food simulant, and temperature. The intended release of AAs from active polymers used for food packaging also depends on the factors previously mentioned. In this context, an important issue in the design of a release system for active food packaging is determining its transport and thermodynamic properties, because these parameters control the release process of AAs from a polymer matrix to the food. Particularly, the release kinetic of the AA should match the food deterioration kinetics. The diffusion coefficient (*D*) is the most relevant parameter to be controlled in active material design, because it characterizes the release kinetics process of AAs from dense polymers to foods. In this context, this section aims to assess the effect of an AA-incorporation technique on its release kinetics and on revising strategies to modify the *D* of AAs in active PLA films without considering polymer blends, composites, and nanocomposites.

Release kinetics parameters will be dependent on the PLA-processing technique and AA properties. Table 5 summarizes some AA diffusion coefficients in PLA films obtained using different processing techniques. Specifically, *D* values of AAs have displayed the lowest values in PLA monolayer films obtained through extrusion/compression molding processing, because these techniques generate PLA films with higher crystallinities than other processing techniques, such as solvent casting and electrospinning. On the other hand, the effect of the AA’s chemical structure also affects *D* values. The development of an antioxidant PLA film with merkén, an aboriginal Chilean spice rich in polyphenols, by extrusion, has reported a *D* value of 2 × 10^−13^ m^2^ s^−1^ (EtOH 95% at 40 °C) [43]. Meanwhile, a 3-fold higher *D* value was reported for thymol in active extruded PLA films (6 × 10^−13^ m^2^ s^−1^; EtOH 95% at 40 °C) [12], which could be explained due to its lower molecular weight than merkén polyphenols.

*D* coefficients of AAs in PLA films were normally higher than *D* values reported in commonly used polymers for food packaging. This fact represents a drawback for the development of release systems based on highly volatile AAs. For example, the *D* value of thymol in extruded PLA films manifested approximately 59-fold higher than the *D* value of thymol in extruded PP films (1× 10^−14^ m^2^ s^−1^; EtOH 95% at 40 °C), which was explained by its lower crystalline degree compared to PP [80].

Active PLA films developed through the solvent-casting process presented lower barrier properties toward mass transfer than extruded films, due to the lower crystallinity and the residual solvent in cast PLA films, which favors the AA’s molecular diffusion through the polymeric matrix. For example, the reported *D* value of thymol in solvent-cast PLA films (2.5 × 10^−13^ m^2^ s^−1^; EtOH 10% at 40 °C) resulted in a value approximately 1.67-fold higher than that reported for extruded PLA films (1.5 × 10^−13^ m^2^ s^−1^; EtOH 10% at 40 °C) [15]. It is important to highlight that thymol was incorporated in both films through an scCO_2_ impregnation process using the same operational conditions.

In recent years, scCO_2_ impregnation has emerged as an innovative technique to incorporate highly thermo-sensitive AAs into PLA films. In this process, active compound diffusion inside a polymer is facilitated by the increase in the polymer chain’s movement of its amorphous zones, due to the plasticizing properties of scCO_2_, and the swelling of its crystalline parts [81]. Nevertheless, the main drawback of this process is that the plasticizing effect of scCO_2_ causes an irreversible decrease in PLA crystallinity. Thus, impregnated PLA films with low degrees of crystallinity allowed for a faster AA diffusion through their structure. This fact entailed a 47-fold increase in the *D* value of thymol in extruded and scCO_2_-impregnated PLA films (2.8 × 10^−11^ m^2^ s^−1^; EtOH 95% at 40 °C) [15] with respect to the value reported for thymol incorporated in PLA by extrusion [12].

**Figure 2:**
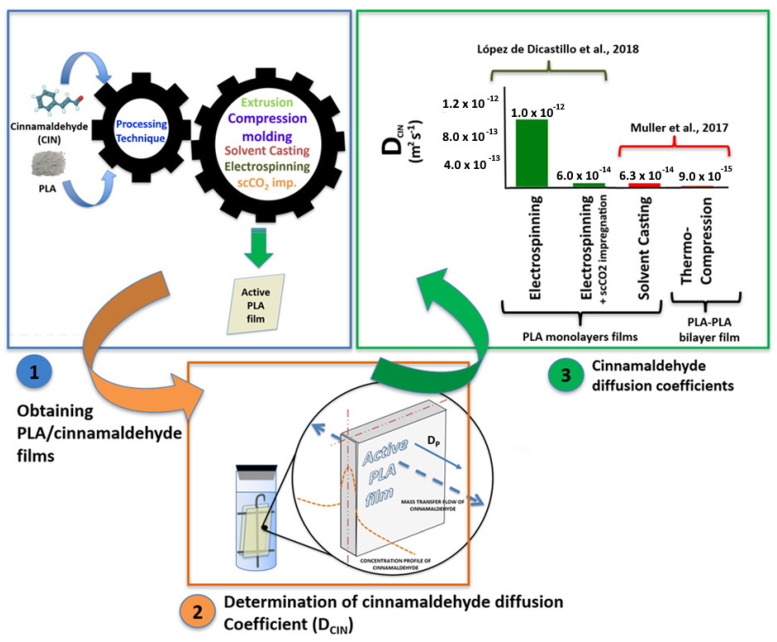
CIN diffusion coefficients into EtOH 50% as food simulant as a function of the processing methods [42,79].

Figure 2 encompasses the results reported of *D* values of CIN, *D_CIN_*, in PLA films obtained using different techniques: electrospun PLA-CIN film, CIN scCO_2_ impregnation of electrospun PLA film, solvent-cast, and compressed molded bilayer films. Interestingly, the scCO_2_ impregnation process showed a different effect on electrospun PLA fibers where an approximately 17-fold reduction in *D_CIN_* value in electrospun PLA film was reported when CIN was incorporated through scCO_2_ impregnation instead of by conventional electrospinning (from 1 × 10^−12^ to 6 × 10^−14^ m^2^ s^−1^; EtOH 50% at 40 °C) [42]. This effect could be related to the fact that sCO_2_ impregnation allowed for the CIN incorporation within the polymer matrix, increasing the diffusion path for CIN release.

Finally, a recent trend to modify the release rate of an AA from a polymer matrix is to perform this through the development of multi- or bi-layer structures. A higher reduction in *D* values can be obtained by using multilayer systems developed through techniques that generate coating layers with high crystallinity (extrusion/compression molding) instead of using techniques that generate layers with low crystallinity (casting and electrospinning). A high reduction (7-fold) in the *D_CIN_* (from 6.3 × 10^−14^ to 9.0 × 10^−15^ m^2^ s^−1^, EtOH 50% at 20 °C) was obtained when a layer of semi-crystalline PLA was deposited by compression molding over an amorphous PLA layer containing CIN [80] (Figure 2).

Meanwhile, a low reduction in the *D* of curcumin (1.3 × 10^−14^ to 0.9 × 10^−14^ m^2^ s^−1^; EtOH 10% at 40 °C) has been reported by adding a low-crystalline layer of PLA on a poly(vinyl alcohol) (PVA) fiber containing curcumin by coaxial electrospinning [14].

The development of active PLA films mainly considers the use of EOs or their derivatives, as well as plants or natural waste extracts. In addition, most of these compounds have been recognized as safe (GRAS) by the Food and Drug Administration, indicating that these substances or compounds have adequately demonstrated their non-toxicity through scientific procedures that require the sufficient quantity and quality of scientific evidence for approval [82,83]. Most of the studies on active PLA films have incorporated AAs at concentrations lower than 10 wt%, and these values have been significant in exhibiting excellent antimicrobial and antioxidant properties. Furthermore, in most cases, the amount of migrated compound into food simulants has not exceeded the FDA’s limits.

Table 6 and Table 7 present recent studies on PLA film developments by the addition of natural antimicrobials and antioxidant agents, respectively. These active films were developed through different techniques, and their functionalities were analyzed by different methods. Direct contact assays, such as disk diffusion or dynamic contact, were the most used methods to analyze the antibacterial activity of active PLA films. The antibacterial activity of PLA-cast films loaded with CIN against *Escherichia coli* and *Listeria innocua* was evaluated by means of the disk diffusion method for 13 days. A bacteriostatic effect against *E. coli* during the first 6 days and a bactericidal effect after this period was reported. However, the growth of *L. innocua* was only inhibited for 9 days due to the progressive evaporation of CIN during the assay time [79]. Likewise, a strong antibacterial activity against *E. coli* and *Staphylococcus aureus* of PLA films, with and without C30B nanoclays, impregnated with thymol or CIN by scCO_2_ impregnation, was obtained by dynamic contact assays [17].

The antimicrobial properties of antimicrobial PLA films were also evaluated without direct contact with the microorganisms. The antifungal properties of PLA films containing beta-cyclodextrin inclusion complexes with thymol and carvacrol (at 1.5, 2.5 and 5 wt%) were evaluated against the *Alternaria alternata* fungus for 10 days through the vapor phase diffusion method. Using this method, mold growth was completely inhibited by PLA films containing the highest concentrations of inclusion complexes containing AAs [52].

Table 7 shows the different methodologies used to measure the antioxidant activity of active PLA films. Assays based on reducing power through electron transference such as FRAP (ferric-reducing ability of plasma) or through free radical scavenging activities such as the DPPH (bleaching rate of stable radical 2,2-diphenyl-1-picrylhydrazyl) and ABTS (inhibition of the cationic radical 2,2-azinobis(3-ethylbenzothiazoline-6-sulfonate)) methods were principally performed.

On the other hand, the Folin–Ciocalteu colorimetric assay has been widely proposed to determine the total phenolic content (PC) of active PLA films. A study developed by Liu et al. (2018) evaluated the antioxidant properties of electrospun PLA films loaded with different amounts of tea polyphenol (TP) through the DPPH method, and reported radical inhibition greater than 70% and proportional to the TP content [58]. Likewise, a recent study incorporated different concentrations of propolis extract in PLA through solvent casting, in order to protect the sausage from oxidation. The PC in the sausage was determined through the Folin–Ciocalteu method, and the PC values increased due to the phenolic constituents released from the active films [22].

After adding the active compound, the study of the durability and stability of PLA packaging films should be considered. However, the scientific information related to this topic is limited. This is due to the fact that most evidence related to the durability of PLA has been mainly associated to thermal decomposition, photo-oxidation, hydrolysis, thermo-oxidation at high temperatures, and natural weathering [1,87]. On the other hand, the monitoring of the functionality of active PLA films over time remains one topic to be considered by researchers for further industrial purposes [88,89].

## 4. Disintegration under Composting

Industrial composting is an option for the disposal of PLA films at the end of their useful life, due to their degradable character at specific conditions. In this context, an important issue in the design of active PLA films for food packaging is determining the effect of the processing technique and of the addition of AAs on their disintegration behavior under composting. The degree of disintegration of plastic films under composting conditions can be determined at laboratory scale by ISO-20200 standard at 58 °C. All the studies reported in this section are in accordance with this ISO.

Under composting conditions, the PLA disintegration process started with the polymer hydrolysis [90], and the disintegration behavior was strongly dependent on the PLA’s physical properties. Investigations [19,20] evidenced that the surfaces of electrospun PLA fibers were more hydrophobic than those of PLA films obtained through other techniques, which tended to delay water sorption and the hydrolysis process (first stage of the disintegration process) [6,91]. The water contact angle (WCA) classifies a surface as hydrophobic when the WCA of the water droplet exceeds 90°. Although electrospun PLA films (115° WCA, 5.7% X_C_) have displayed lower thickness and crystallinity (X_C_) than melt-blending-based PLA films (58° WCA, 15.7% X_C_), Arrieta et al. have reported a 28-day period for the total disintegration of both processed PLA films [19,20].

On the other hand, although PLA films obtained by extrusion and melt blending present a similar degree of hydrophilicity, extruded PLA films required the shortest times for their total disintegration due to their lower X_C_ values. Villegas et al. (2019) recently reported 14 days for the total disintegration of extruded PLA films (77 μm, X_C_ 4%, WCA 78°) [17]. The thickness has also been reported as an important factor, which influenced the time required for the whole disintegration of PLA films. Only seven days were reported for the whole disintegration of extruded PLA films with 35 µm-thick [66]. Meanwhile, 57 days were necessary for the complete disintegration of extruded PLA films with higher thicknesses [92].

The effect of the incorporation of an AA on the total disintegration time of PLA depended on its accelerating or delaying role in the process. The addition of an AA that plasticizes PLA sped up the first stage of its disintegration process due to an increase in the mobility of the polymer chains, which allowed a faster water diffusion inside the polymeric matrix. The well-known plasticizing effect of thymol on PLA and the presence of hydroxyl groups in the thymol structure, which improved the water absorption, were reported as the main factors that entailed the speed-up of the heterogeneous hydrolysis of active extruded PLA films. The absorbed water resulted in the formation of labile bonds in the PLA structure, increasing the disintegration rate [66,80,93]. This acceleration during the first stage of the PLA disintegration process was also observed when CIN was incorporated in extruded films through scCO_2_ impregnation [94]. Moreover, the disintegration of PLA is highly dependent on the affinity between the AA and water, which modifies the hydrophilicity of PLA. The higher degree of plasticization and hydrophilicity of extruded PLA films due to their impregnation with thymol (20.5 wt%) instead of CIN (13 wt%) were related to the shortest half-maximal disintegration times reported for PLA with thymol (5.98 days) than with CIN (6.35 days), compared to the neat PLA film (6.83 days) [17].

In some cases, the AA presented nucleating properties and, therefore, their incorporation delayed the disintegration process due to the increase of polymer crystallinity, which hindered both the hydrolysis of the polymer and the microorganism’s enzymatic attack (second stage of the disintegration process). The nucleating properties of catechin at 5 wt% in electrospun PLA/PHB films increased the disintegration time from 28 to 37 days, even considering that the first stage of the disintegration process was sped up because the catechin increased the hydrophilicity of the film [95]. Limonene also exhibited a nucleating effect in melt-blending-based PLA films, which counteracted the acceleration of the first stage of the disintegration process caused by the plasticizing effect of limonene, reaching a similar time (28 days) to that reported for the whole disintegration of neat PLA [19].

The incorporation of AAs with antibacterial activities in PLA films delayed the second stage of its disintegration process due to the inhibition of the microorganism action that converts the small molecules produced by PLA hydrolysis into CO_2_ and water. Iglesias-Montes et al. (2021) reported a delay in the second stage of the disintegration process of PLA due to the addition of UMB at 15 wt% [96]. In other work, thymol at 6 and 8 wt% in extruded PLA films delayed the second stage of the disintegration process of PLA, counteracting the acceleration of the hydrolysis caused by the plasticizing effect of this AA [66,92]. Similarly, extruded active PLA films obtained by sCO_2_ impregnation with thymol at 20.5 wt% also delayed the second stage of the disintegration process compared with the neat PLA film [17].

## 5. Conclusions and Future Perspectives

In recent decades, the use of PLA to design active films for food packaging by incorporating AAs through different processing techniques has been explored extensively. The color and opacity of PLA films have been the main optical properties affected by the incorporation of AAs. Notable color changes, as well as the increase of the opacity in these materials, have been the most highlighted results. The thermal properties of PLA varied with their processing conditions due to their influence on the chain ordering. Furthermore, when an AA was added to PLA, the thermal properties of the resulting active PLA films depended on the molecular structure of the AA, its nucleating and/or plasticizing effect on the polymer structure, its concentration, and the dispersion degree reached from each processing technique. It was highlighted that the thermal properties of active PLA produced through melting-based and solvent-cast processes were highly dependent on the temperature of processing and solvent volatility, respectively. Regarding mechanical properties, the addition of the AA decreased the elastic modulus of PLA for all processing techniques attributed to a plasticizer effect of the AA, with the exception of the addition of chitosan until 10 wt% as polymer reinforcements, because of the rigidity of the polysaccharide chains and the generated interactions between PLA and AA. In addition, the tensile strength was changed as a function of component interactions, dispersion, and concentration of the AA. Meanwhile, the elongation at break was increased when the AA presented a plasticizing effect, and was reduced due to a lack of homogeneity or film discontinuities.

On the other hand, low AA concentration has allowed for high microbial growth inhibition and strong antioxidant activity of active PLA films, the most used AAs being essential oils and their derivatives, and plant extracts. Mostly, the concentration of the AA used was lower than 10 wt%. The processing and environmental conditions and overtime are the main factors that can affect the antimicrobial and antioxidant properties of active PLA films. For this reason, the evaluation of the food’s shelf life is an important topic to be considered in future investigations.

Release properties of PLA were highly dependent on the physical properties and crystallinity resulting from the different processing techniques. In this context, it is mainly emphasized that the AA displayed higher diffusion coefficients in PLA films obtained by solvent casting than those obtained in melting-based processed PLA films, due to the residual solvent in the cast PLA film and its lower crystallinity. Conversely, the disintegration behavior of PLA under composting varied as a function of surface wettability, crystallinity, and the thickness of the PLA film obtained from each processing technique. The addition of an AA induced changes in the physical properties of PLA, and these properties governed its compostability, depending on the chemical nature and amount of the AA added. In general, AAs that plasticized PLA tended to accelerate the PLA’s disintegration process. Meanwhile, AAs with nucleating properties generally delayed the disintegration process of PLA.

In addition to PLA composting, a second possible end-of-life option for these materials is recycling, but there is a lack of information about it. The study of the effect of incorporating AAs into PLA on its recyclability is surely an important issue to be taken into account for further research, considering how the technology of active packaging based on PLA could potentially contribute to a reduction in plastic and food waste.

Regarding future perspectives, the major concern in developing active PLA food packaging is the lack of connection with current regulations. Any AA added to a food packaging system must comply with migration values under the regulatory limits and concentrations that prevent sensory changes in the product. Thus, a general challenge for active packaging is finding new strategies to reduce the amount of an AA and to maintain an adequate activity, as well as to keep migration levels below the regulated limits. In this context, a future direction to solve this problem could be the use of the well-known synergistic effect of natural compounds, which could allow the reduction of the necessary AA concentration. A second strategy could be to consider non-migratory active PLA packaging films by means of the attachment of the AA through covalent immobilization onto the PLA surface.

## Figures and Tables

**Table 1:** Thermal properties of PLA films obtained through different processing techniques.

PLA							
Processing Technique	PLA	Processing Condition *	T_g_ (°C)	T_m_ (°C)	X_c_ (%)	T_d_ (°C)	References
Cast-extrusion	NW2003D	Temperature: 170–185 °C	61.7	170.8	19.5	362.0	[37]
	NW **	Temperature: 155–165 °C	63.2	155.6	4.6	365.1	[43]
Electro-spinning	NW2003D	Solvent: CHCl_3_:DMF 1:1	53.1	153.2	1.1	334.0	[42]
Casting	NW **	Solvent: Methylene chloride Evaporation: 25 °C for 24 h under a chemical hood	59.7	168.4	N/R	N/R	[44]
	NW2002D	Solvent: Chloroform Evaporation: room temperature and 3 days under a chemical hood	44.3	166.4	N/R	N/R	[45]

(*) Some processing conditions were included for a better understanding of comparisons among works explained in Section 2.2; (**) NW: PLA provided by NatureWorks was the only information reported. The maximum decomposition temperature (T_d_) was determined by thermogravimetric analysis (TGA).

**Table 2:** Thermal properties of active PLA films obtained through different processing techniques.

Active PLA Films								
Processing Technique	Active Agent/Condition	PLA	AA Composition (wt%)	T_g_ (°C)	T_m_ (°C)	X_c_ (%)	T_d_ (°C)	References
Casting	*Allium ursinum* extract Solvent: CHCl_3_	Esun	0	42.6	147.9; 158.5	7.3	N/R	[31]
			0.5	45.9	143.6; 155.6	13.2		
			5	45.5	149.6; 159.2	6.4		
	Bergamot and clove oil	NW *	0	55.0	166.7	11.7	361	[49]
			9% Bergamot in CHCl_3_	52.0	164.5	13.0	58–173; 361	
			9% Clove in CHCl_3_	49.2	159.6	16.6	76–186; 363	
Electro-spinning	Curcumin	NW2003D	0	40.2	136.9; 147.3	21	331.5	[14]
			1.23% in solution CHCl_3_:DMF 7:3	54.7	149.2	6.2	353.6	
	Ungeremine	NW4042D	0	59.3	148.6	4.1	N/R	[47]
			1	54.2	147.8	8.53		
Extrusion-biaxial stretching	Thymol or R-(−)-carvone	NW4043D	0	57.0	150.0	27.6	N/R	[48]
			20 wt% thymol	56.0	140.0	13.6	N/R	
			20 wt% R-(−)-carvone	54.0	144.0	34.9	N/R	
Injection **	Thymol	PLI005	0	61	154.0	31.0	367	[38]
			5	56	154.0	29.0	N/R	
			20	35	135.0	33.0	117–300; 1366	

(*) NW: PLA provided by NatureWorks was the only information reported; (**) Results correspond to the second DSC heating process; all others correspond to the first DSC heating. T_d_: maximum decomposition temperature of PLA, but in cases where a temperature range followed by another value are reported, the temperature range corresponds to the gradual evaporation of the essential oil moisture or degradation of the AA, and the last value corresponds to the maximum decomposition temperature of PLA. N/R: information non-reported.

**Table 3:** Effect of natural active agent incorporation on mechanical properties of PLA films processed through different techniques.

Processing Technique	PLA/Active Agent	YM (MPa)	TS (MPa)	EB (%)	References
Melt blending-hot pressing	PLA control	3361	56.82	2.21	[32]
	Waste orange peel extract Rosemary ethanolic extract with or without chitosan	↓	↓	≈ or ↑	[32,55]
	Chitosan	↑	↑	↓	[55]
Cast extrusion	PLA control	3000	65	3.1	[51]
	*Allium* spp. Extract Oregano essential oil Merkén	↓	↓	↓	[43,51,59]
Extrusion-stretching	PLA control	-	-	40	[48]
	Thymol, R-(−)-carvone	↓	↓	↑	
Blown extrusion	PLA control	-	40.2	3.63	[60]
	Green tea extract	↓	↓	↑	
Injection molding	PLA control	3100	52.7	2.9	[38]
	Thymol and Carvacrol β-CD-thymol and β-CD-carvacrol	↓	↓	↑	[38,52]
Casting	PLA control	1702	45.8	5.4	[49]
	Clove essential oil Bergamot, lemongrass, rosemary and clove Ethanolic extract of propolis	↓	↓	↑	[13,49,61]
	Allyl isothyocianate Wild garlic	↓	≈ or ↑	≈ or ↓	[31,44]
Electrospinning	PLA control	-	12.24	57.28	[58]
	Tea polyphenol Propolis powder extract	↓	↓	↓	[58,62]
	Propyl gallate	↓	↑	↑	[57]
Supercritical impregnation	PLA control	1607	47.9	3.4	[46]
	Thymol	↓	↓	↑	[15,46]
	Cinnamaldehyde	≈	↓	↑	[17,18]

YM: Young’s modulus. TS: tensile strength. EB: elongation at break. Trends: ↑ (increase), ↓ (decrease), ≈ (invariable). The hyphen indicates that the mechanical parameter or its units were not reported.

**Table 4:** Water vapor and oxygen permeability values of active PLA films processed through different techniques.

Processing Technique	AA	Thick (µm)	X_c_ (%)	PH_2_O	PO_2_	References
Casting	Clove oil (CLO) 15–30 (wt%)	65–70	PLA: 15.58; PLA.15CLO: 14.39; PLA.30CLO: 8.23	N/R	PLA: 16.3; PLA-15CLO: 20.5; PLA.30CLO: 26.6	[13]
	Allyl isoctiocyanate (AIT) 5 (wt%)	N/R	PLA: 20.97; PLA.5AIT: 17.29	PLA: 2.38 PLA.5AIT: 1.93 × 10^−6^ kg m^−2^ s^−1^	PLA: 3.77 ×10^−6^; PLA.5AIT: 2.3 × 10^−6^ (m^3^ m^−2^ s^−1^)	[44]
	Oregano essential oil (EO) 0.5, 1 and 1.5 (wt%)	N/R	N/R	PLA: 1.89; PLA.0.5EO: 1.25; PLA.1.5EO: 1.72 × 10^−8^ kg m m^−2^ s^−1^ Pa^−1^	N/R	[45]
	Bergamot (BEO), lemongrass (LEO), rosemary (REO), clove CEO) 9 (wt%)	N/R	PLA: 11.7; PLA.9BEO: 13.0; PLA.9LEO: 16.3; PLA.9REO: 15.5; PLA.9CEO: 16.6	PLA: 1.06; PLA.9BEO: 2.03; PLA.9LEO: 1.69; PLA.9REO: 1.54; PLA.9CEO: 1.91 × 10^−14^ kg m m^−2^ s^−1^ Pa^−1^	N/R	[49]
	Garlic extract powder (AU) 0.5 and 5 (wt%)	N/R	PLA: 7.3; PLA.0.5AU:13.2; PLA. 5AU: 6.4	PLA: 6.84; PLA.0.5AU: 6.12; PLA.5AU: 5.47 × 10^−15^ kg m^−1^ s^−1^ Pa^−1^	RH = 0%: PLA: 4.8; PLA.0.5AU: 3.84; PLA.5AU: 3.84; RH = 50%: PLA.5GE: 6.08 × 10^−18^ kg m^−1^ s^−1^ Pa^−1^	[31]
Coating	Zein-Quercetin (ZN-Q) 5 (wt%)	N/R	N/R	PLA/ZN: 1.66; PLA/ZN-5Q: 1.83 × 10^−14^ kg m m^−2^ s^−1^ Pa^−1^	N/R	[33]
	Pickering emulsion-Thymol (PE-T) 20–40 (wt%)	N/R	N/R	PLA: 2.43; PLA/PE-T: 3.98 × 10^−14^ kg m m^−2^ s Pa^−1^	PLA: 4.03; PLA/PE-T: 2.07 × 10^−18^m^3^ m m^−2^ s^−1^ Pa^−1^	[70]
Extrusion	Merkén (M) 3 and 5 (wt%)	85–100	PLA: 4.6; PLA.3M: 2.1; PLA.5M: 1.7	PLA: 2.50; PLA.3M: 3.04; PLA.5M: 3.83 × 10^−15^ kg m m^−2^ s^−1^ Pa^−1^	N/R	[43]
	Thymol (T) & R-(−)-carvone oil (C) 10, 15 and 20 (wt%)	30–45	PLA: 27.6; PLA.10T: 25.6; PLA.15T: 16.4; PLA.20T: 13.6; PLA.10C: 32.3; PLA.15C: 32.5; PLA.20C: 34.9	N/R	PLA: 21; PLA.10T: 17; PLA.15T: 24; PLA.20T: 50; PLA.10C: 20; PLA.15C: 18; PLA.20C: 22	[48]
	Green tea e×tract (GT) 1 and 2 (wt%)	47–78	N/R	PLA: 9.31; PLA.1GT: 7.31; PLA.2GT: 6.0 × 10^−7^ kg m^−2^ s^−1^	N/R	[60]
Compression-molding	Gelatin-Epigallocatechin gallate (Gel-EGCG) 3, 6, 9 and 12 (wt%)	179–195	N/R	PLA/Gel:2.01; PLA/Gel.3EGCG: 2.06; PLA/Gel.6EGCG: 2.01; PLA/Gel.9EGCG: 2.02; PLA/Gel.12EGCG: 2.15 × 10^−14^ kg m^−1^ s^−1^ Pa^−1^	N/R	[34]
Electro-spinning	Garlic extract (AU) 10 (wt%)	1–2	PLA: 13.2; PLA.10AU: 7.9	PLA: 9.21; PLA.10AU: 6.9 × 10^−14^ kg m m^−2^ s^−1^ Pa^−1^	N/R	[65]
	Soy protein + HPMC + PEG nanofibers (SP/HPMC/PEG) 33.3/33.3/33.33 (wt%)	31	N/R	PLA: 2.6; PLA/SP-HPMC-PEG: 4.869 × 10^−14^ kg m^−1^ s^−1^ Pa^−1^	N/R	[71]

TBC: tributyl citrate; HPMC: hydroxypropyl methylcellulose; PEG: polyethylene glycol; Pickering emulsion (PE) containing maize germ oil and zein/chitosan complex particles [70,71]; N/R: information non-reported.

**Table 5:** AA Diffusion coefficients in PLA films obtained using different processing techniques.

Active Agent	Processing Technique	Release Conditions	Diffusion Coefficient (m^2^ s^−1^)	References
Merkén	Extrusion	EtOH 50% at 40 °C	2.0 × 10^−13^	[43]
Thymol	Extrusion	EtOH 95% at 40 °C	6.0 × 10^−13^	[12]
	Solvent Casting	EtOH 10% at 40 °C	2.5 × 10^−13^	[46]
	Supercritical impregnation	EtOH 10% at 40 °C	1.5 × 10^−13^	[15]
		EtOH 95% at 40 °C	2.8 × 10^−11^	
Cinnamaldehyde	Electrospinning	EtOH 50% at 40 °C	1.0 × 10^−12^	[42]
	Supercriticalimpregnation		6.0 × 10^−14^	
	Casting (PLA monolayer)	EtOH 50% at 20°C	6.3 × 10^−14^	[79]
	Thermo-compression (PLA-bilayer)		9.0 × 10^−15^	

**Table 6:** Recent studies on antimicrobial properties of PLA-based packaging films.

Active Agent	Processing Technique	Methodology	Antimicrobial Activity	References
Carvacrol and thymol	Extrusion	* Disk diffusion	*S. aureus*: 1.5–2.5; *S. Thyphimurium*: 0.7–0.8; *L. monocytogenes*: 0.9–0.8	[38]
*Allium* spp. extract	Cast Extrusion	** Dilution method	Bacteria: 0–4.9; Moulds: 0–3.9; Yeats: 0–3.9	[51]
β-CD inclusion complexes of thymol and carvacrol	Injection molding	* Vapor phase diffusion	*A. alternate:* 0–29.7 (5 days) and 0–69 (10 days)	[52]
Cinnamaldehyde	Casting	** Disk diffusion	*L. innocua*: 0–4; *E. coli*: 1–7	[79]
Bergamot, lemongrass, clove and rosemary essential oils	Casting	** Liquid culture test	*E. coli*: 2–3; *B. subtilis*: 3.5.	[49]
*Copaifera multijuga* oil	Coating	* Disk diffusion	*B. subtilis*: 20	[21]
Rosemary, caraway and fennel oils	Coating	Normative ASTM E 2180-07	*S. aureus*: 81–85%; *E. coli*: 66–70%	[84]
Propolis extract	Electrospinning	Direct contact	*S. aureus*, *E. coli*, *S. epidermis*, *B. cereus; P. mirabilis:* Bactericidal effect (4 wt%)	[62]
*Allium ursinum* L. extract	Electrospinning	** Direct contact	*E. coli*: 5.9; *S. aureus*: 2.1	[65]
Cinnamaldehyde	scCO_2_ impregnation	Dynamic contact	*E. coli*; *S. aureus*: total inhibition	[18]
Thymol and CIN	scCO_2_ impregnation	Dynamic contact	*E. coli*; *S. aureus:* total inhibition	[17]

Results were expressed as: * Measurement of inhibition diameter in mm, and ** Logarithmic reduction.

**Table 7:** Recent studies on antioxidant properties of PLA-based packaging films.

Active Agent	Processing Technique	Methodology	Activity	References
Green tea (GT) extract	Extrusion	Packaged salmon analysis: PV (peroxides value), p-anisidine value, TBARS	Salmon packaged for 60 days: PV: no detected; p-anisidine and TBARS: 33% reduction on aldehydes	[60]
Propolis extract	Casting	Packaged sausage analysis: Folin–Ciocalteu	Sausage packaged for 4 days: PC between 0.6 and 1.7 ***	[22]
Bergamot essential oil	Casting	Packaged mangoes analysis: Vitamin C quantification	Mangoes packaged for 15 days: Vitamin C was maintained between 42% to 75%	[85]
Tea polyphenol	Electrospinning	DPPH	DPPH inhibition: 70 to 95%	[58]
HPβ-CD inclusion complexes of gallic acid	Electrospinning	DPPH	DPPH inhibition higher than 95%	[86]

Results were expressed as: *** mg of gallic acid/g sample. PC: Total polyphenol content; DPPH: bleaching rate of radical stable 2,2-diphenyl-1-picrylhydrazyl; TBARS: thiobarbituric acid reactive substances assay.
